# Cellular Immunity of *Drosophila willistoni* Reveals Novel Complexity in Insect Anti-Parasitoid Defense

**DOI:** 10.3390/cells13070593

**Published:** 2024-03-29

**Authors:** Gyöngyi Cinege, Kinga Fodor, Lilla B. Magyar, Zoltán Lipinszki, Dan Hultmark, István Andó

**Affiliations:** 1 Innate Immunity Group, Institute of Genetics, HUN-REN Biological Research Centre, 6726 Szeged, Hungary; kingafodor02@gmail.com (K.F.); magyar.lilla@brc.hu (L.B.M.); ando@brc.hu (I.A.); 2 Doctoral School of Biology, University of Szeged, 6720 Szeged, Hungary; 3 MTA SZBK Lendület Laboratory of Cell Cycle Regulation, Institute of Biochemistry, HUN-REN Biological Research Centre, 6726 Szeged, Hungary; lipinszki.zoltan@brc.hu; 4 National Laboratory for Biotechnology, Institute of Genetics, HUN-REN Biological Research Centre, 6726 Szeged, Hungary; 5 Department of Molecular Biology, Umea University, 901 87 Umea, Sweden; dan.hultmark@umu.se

**Keywords:** hemocyte, multinucleated, immune response, *Drosophila willistoni*, parasitoid wasp, immune cell, phagocytosis, encapsulation, giant cell

## Abstract

Coevolution of hosts and their parasites has shaped heterogeneity of effector hemocyte types, providing immune defense reactions with variable effectiveness. In this work, we characterize hemocytes of *Drosophila willistoni*, a species that has evolved a cellular immune system with extensive variation and a high degree of plasticity. Monoclonal antibodies were raised and used in indirect immunofluorescence experiments to characterize hemocyte subpopulations, follow their functional features and differentiation. Pagocytosis and parasitization assays were used to determine the functional characteristics of hemocyte types. Samples were visualized using confocal and epifluorescence microscopy. We identified a new multinucleated giant hemocyte (MGH) type, which differentiates in the course of the cellular immune response to parasitoids. These cells differentiate in the circulation through nuclear division and cell fusion, and can also be derived from the central hematopoietic organ, the lymph gland. They have a binary function as they take up bacteria by phagocytosis and are involved in the encapsulation and elimination of the parasitoid. Here, we show that, in response to large foreign particles, such as parasitoids, MGHs differentiate, have a binary function and contribute to a highly effective cellular immune response, similar to the foreign body giant cells of vertebrates.

## 1. Introduction

Insect hemocytes constitute the cellular elements of a powerful innate immune response that shows many similarities to the innate immune system of vertebrates [1,2,3]. Our understanding of insect and vertebrate innate immunity largely comes from the analysis of the model organism, *Drosophila melanogaster*. Hemocytes of *D. melanogaster* phagocytose microbes, produce antimicrobial peptides, encapsulate parasitoids and contribute to tissue remodeling during development. These processes are generated by three main hemocyte classes: the plasmatocytes, the crystal cells and the lamellocytes, which are localized in three hematopoietic compartments: the circulation, the lymph gland and the sessile tissue [4,5,6]. The most prevalent cells in the circulation are the plasmatocytes. They are spherical cells that phagocytose microbes, produce antimicrobial peptides and matrix proteins, and contribute to the elimination of parasitoids as they attach to the chorion at the beginning of capsule formation [7]. After cuticle damage or following parasitoid infection, plasmatocytes transdifferentiate into large, flattened non-phagocytic, encapsulating cells, termed lamellocytes [4,8]. Lamellocytes differentiate in circulation, sessile tissue and the main hematopoietic organ, the lymph gland [9]. The encapsulation reaction starts with the attachment of plasmatocytes to the parasitoids; then, lamellocytes form several cellular layers around eggs and larvae of the wasp to envelop and isolate the developing intruder [7]. Lamellocytes, the characteristic encapsulating cell type in the species of the *melanogaster* subgroup, produce prophenoloxidase 3 (PPO3), a key enzyme in the melanization of the capsule and in killing the parasitoid [10]. The crystal cells carry prophenoloxydases and represent a minor subpopulation of hemocytes [11,12]. These cells contain components of the melanization system that is activated upon wounding and the encapsulation reaction. Based on transcriptomic studies in *D. melanogaster*, a new class of hemocytes, called primocytes, have been identified. These cells are present in every hematopoietic compartment and are likely to be involved in the activation of effector hemocytes [13,14].

Certain non-model species of *Drosophilidae* utilize other effector hemocyte types to encapsulate parasitoids. Non-phagocytic, multicellular, web-like structures have been found in species of the *ananassae* subgroup, in *Zaprionus indianus*, *Drosophila falleni*, *Drosophila phalerata* and *Drosophila grimshawi* [15,16] upon infection by a variety of parasitoid wasps. Species differentiating multinucleated hemocytes are highly resistant to endoparasitoids [15,17]. Multinucleation provides a metabolic advantage for the cells as the increased genetic material within one cytoplasm leads to higher protein expression levels and intensified metabolism [18,19], which may also contribute to the effective anti-parasitoid immune response [15,17]. Hemocytes with more than one nucleus have been described in naïve *Drosophila willistoni* and were classified as plasmatocytes [20]. In this species, hemocytes have been grouped on the basis of their morphological features as plasmatocytes, podocytes, spheroidocytes, oenocytoids, nematocytes and crystalloid cells [20].

The aim of the present study was to systematically group and characterize hemocytes of *D. willistoni* by a combination of immunological and functional analyses. In particular, our focus is directed toward the multinucleated hemocyte population, and we assess their function in terms of defense against endoparasitoid wasps.

## 2. Materials and Methods

### 2.1. Insect Stocks and Culturing

*D. willistoni* wild type (14030-0811.24) was obtained from UC San Diego *Drosophila* stock center. *D. melanogaster* wild type (Oregon-R) was acquired from Bloomington Drosophila Stock Center. Each strain was maintained at 25 °C on standard yeast-cornmeal food. The *Leptopilina boulardi* (G486), *Leptopilina heterotoma* 14 and *Leptopilina victoriae* UNK parasitoid wasps were provided by Prof. Todd Schlenke (University of Arizona, Tucson, AZ, USA) and maintained on *D. melanogaster* wild type Oregon-R. Wasps were kept at 25 °C on 1.5% agar supplemented with 10% glucose, with additional honey placed on blotting-paper.

### 2.2. Generation of Wasp-Induced Samples and Parasitization Assay

A total of 60 early second instar *D. melanogaster* or *D. willistoni* larvae were exposed to 15 female *Leptopilina boulardi*, *Leptopilina heterotoma* or *Leptopilina victoriae* parasitoid wasps for 6 h at 25 °C. Then, 48 h after the infection, the parasitized larvae were selected under dissecting microscope based on the melanized sites of the oviposition, showing that the respective larva was subjected to successful parasitoid attack. The selected infected larvae were used in the respective experiments.

To test parasitization of *D. willistoni* and *D. melanogaster* by the three parasitoid wasp species, four independent experiments were carried out; one representative image is presented. To generate the 1:1 ratio of *D. melanogaster* and *D. willistoni* larvae in the respective parasitization experiments, we applied 30-30 early second instar larvae of both fly species into the vials.

For parasitization assay 48 h after the infection, 10 of the 60 infected larvae were dissected to test the parasitization success, and a vial was considered available for the assay when each larva carried at least one parasitoid [17]. For each combination, the results of four independent experiments were cropped.

### 2.3. Production of Monoclonal Antibodies

A total of 3 one-month-old female BALB/C mice were immunized three times at 3-week intervals with 10^5^ hemocytes isolated in PBS from *L. victoriae* parasitoid wasp infected *D. willistoni* larvae. For hybridoma generation, the spleen cells were fused with Sp2/0 myeloma cells using polyethylene glycol (PEG 1450). Hybridomas were maintained and selected by Kohler and Milstein’s hybridoma technology (1976). Supernatants of individual cultures were screened by indirect immunofluorescence, and positive clones were maintained further. The hybridoma lines producing antibodies reacting with a fraction of cells or with all hemocytes were cloned at 0.3 cell/culture to ensure homogeneity. Tissue culture supernatants of individual clones were selected, including three discriminative (10C5, 1C1 and 8G5) and one panhemocyte antibody (1F1).

### 2.4. Hemocyte and Parasitoid Isolation, Preparation of the Lymph Gland

All procedures were carried out at room temperature. Individual *D. willistoni* larvae were dissected in Schneider’s medium (Lonza, Basel, Switzerland) supplemented with 5% fetal bovine serum (GIBCO, Grand Island, NY, USA) and 0.01% 1-phenyl-2-thiourea (Sigma, St. Louis, MO, USA) (CSM) in 30 µL volume on multispot microscope slides (Hendley-Essex, Loughton, UK). The released hemocytes were allowed to settle and adhere to the spots for 1 h, and then were processed further, as described in Section 2.5. Parasitoids were harvested by micropipette from the hemolymph of the dissected larvae and processed for immunostaining. The lymph glands were prepared from wasp-infected larvae 24 h after parasitization and from age matched naïve animals. The larvae were dissected along the longitudinal axis on the ventral part, and the digestive tract and fat body were removed.

### 2.5. Indirect Immunofluorescence and Image Analysis

The adhered hemocytes were fixed for 6 min with acetone, dried for 3 min, and blocked with PBS supplemented with 0.1% BSA for 20 min. The parasitoids and lymph glands were fixed with 2% paraformaldehyde for 10 min, blocked and permeabilized with 0.1% BSA in PBS supplemented with 0.1% Triton X-100. Samples were incubated with the respective hemocyte specific primary monoclonal antibodies in the form of undiluted hybridoma supernatants (1F1, 10C5, 1C1 or 8G5) and T2/48 as negative control. The anti-phospho-histoneH3 (aH3P) was a rabbit polyclonal primary antibody (Sigma, 1:1000 dilution), and the anti-bromodeoxyuridine (aBrdU) was an Alexa Fluor 488-conjugated mouse monoclonal primary antibody (Invitrogen (Waltham, MA, USA), 1:10 dilution). Samples were washed 3 times for 5 min in PBS, and then incubated with the secondary antibodies and DAPI for 45 min. Secondary antibodies were: anti-mouse Alexa Fluor 488 goat antibody (Invitrogen, 1:1000 dilution), anti-mouse CF 568 goat antibody (Biotium (Fremont, CA, USA), 1:1000 dilution) and anti-rabbit Alexa Fluor 568 goat antibody (Invitrogen, 1:1000 dilution). Samples were washed 3 times with PBS for 5 min and covered with Fluoromount G medium and coverslip. Analysis was performed with an Olympus (Tokyo, Japan) FV1000 confocal LSM microscope or an epifluorescence microscope (Zeiss Axioscope (Jena, Germany) 2 MOT). Hemocytes were counted with the ImageJ program (version 1.52a) based on nuclear DAPI fluorescence.

### 2.6. BrdU Labeling of the Larvae and Ex Vivo Detection of Cell Fusion

The flies laid eggs in vials containing standard fly food supplemented with 1 mM BrdU, in the dark. Second instar larvae were parasitized by *L. victoria*e for 6 h in the dark. Hemocytes were isolated from the larvae 48 h after parasitization. Hemocytes of BrdU-treated and untreated larvae were mixed in CSM, then allowed to settle at room temperature for 1 h on a microscopic slide. The attached cells were fixed with acetone for 6 min, treated with 2 M HCl for 15 min, washed 4 times in PBS, and blocked in PBS containing 0.1% BSA for 20 min. As primary reagent, the mouse anti-BrdU Alexa Fluor 488-conjugated antibody was used for 1 h, then the samples were washed 3 times in PBS, and anti-mouse Alexa Fluor 488 goat antibody (Invitrogen, 1:1000 dilution) was applied for 45 min. Nuclei were visualized with DAPI (Sigma, 1:400). The slides were washed 3 times in PBS, covered with Fluoromount G medium and coverslip, then analyzed with an epifluorescence microscope (Zeiss Axioskope 2 MOT).

### 2.7. Phagocytosis Assays

Fluorescein isothiocyanate (FITC)-labeled *Escherichia coli* bacteria (SzMC 0582) (Szeged Microbial Collection, University of Szeged, Szeged, Hungary) were used. For ex vivo phagocytosis assay, hemocytes of 4 larvae were pooled in 100 μL CSM. Six μL *E. coli*-FITC conjugate was added from a 10% bacterial suspension in sterile PBS to the hemocytes, which were incubated for 45 min at room temperature. The fluorescence of the non-phagocytosed bacteria was quenched with trypan blue in 0.2% final concentration. Fluorescence of the engulfed bacteria was visualized with an epifluorescence microscope (Zeiss Axioscope 2 MOT). The in vivo phagocytosis assay was carried out by injecting naïve or infected larvae (72 h after parasitization) with 0.1 μL *E. coli*-FITC from a 4% bacterial suspension in sterile PBS. Injected larvae were incubated at room temperature for 1 h under sterile conditions in petri dishes on Whatman paper steeped with *Drosophila* Ringer solution. Hemocytes were isolated, adhered on microscopic slides for 1 h, fixed in acetone and reacted with the respective antibodies as described above for indirect immunofluorescence analysis. To detect phagocytic ability of hemocytes settled on the parasitoids, 42 µL of 10% FITC-labeled bacteria was added to 700 µL CSM containing the isolated parasitoids, mixed and incubated for 1 h. Then, parasitoids were washed 3 times with PBS, and indirect immunofluorescence analysis was performed as described above. The samples were analyzed with an Olympus FV1000 confocal LSM microscope, and images in the nucleus plane were used.

## 3. Results

### 3.1. Efficiency of Parasitization

**Figure 1:**
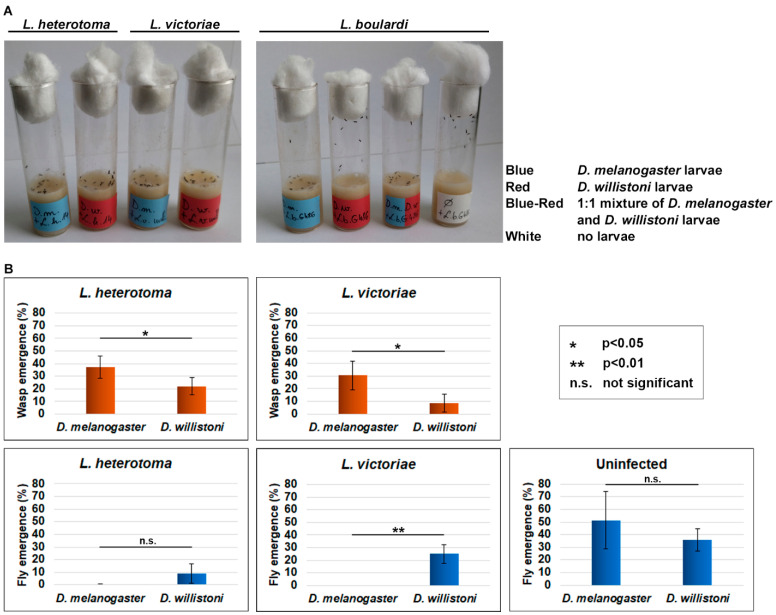
Parasitization of *D. willistoni* and *D. melanogaster.* (**A**) *L. boulardi* do not parasitize *D. willistoni* larvae. The image is representative of four independent experiments. (**B**) Eclosion success of *D. willistoni* was compared to that of *D. melanogaster* following infection with *L. heterotoma* and *L. victoriae* parasitoids. Four independent experiments were carried out with 50 larvae in each. The error bars indicate the standard deviation. Student’s *t*-test was used for statistical analysis.

Several parasitoid wasp species lay their eggs in *Drosophila* larvae. In this work, to examine the immune defense reactions of *Drosophila willistoni*, we used *Leptopilina heterotoma*, *Leptopilina victoriae* and *Leptopilina boulardi* parasitoid wasps and related the parasitization efficiency to that of *Drosophila melanogaster*. We observed that *D. melanogaster* larvae attracted all three wasp species, while *D. willistoni* was parasitized only by *L. heterotoma* and *L. victoriae* (Figure 1A). A 1:1 mixture of *D. melanogaster* and *D. willistoni* attracted *L. boulardi* wasps (Figure 1A, second image). In the medium control, parasitoids showed dispersed arrangement near the cotton wool stopper on the wall of the vial, as in the *D. willistoni*-*L. boulardi* combination.

As *L. boulardi* did not attack *D. willistoni*, the wasp and fly eclosion rates were analyzed following infection with *L. heterotoma* and *L. victoriae*. The eclosion rates for both parasitoid wasp species were significantly lower in *D. willistoni* than in *D. melanogaster* (Figure 1B, upper panel). This suggests that a more efficient killing mechanism operates in *D. willistoni* than in *D. melanogaster*. Furthermore, *D. willistoni* was less likely to survive parasitization by *L. heterotoma* than by *L. victoriae* (Figure 1B, lower panel).

### 3.2. The Cellular Immune Response of D. willistoni to Parasitoid Wasps

**Figure 2:**
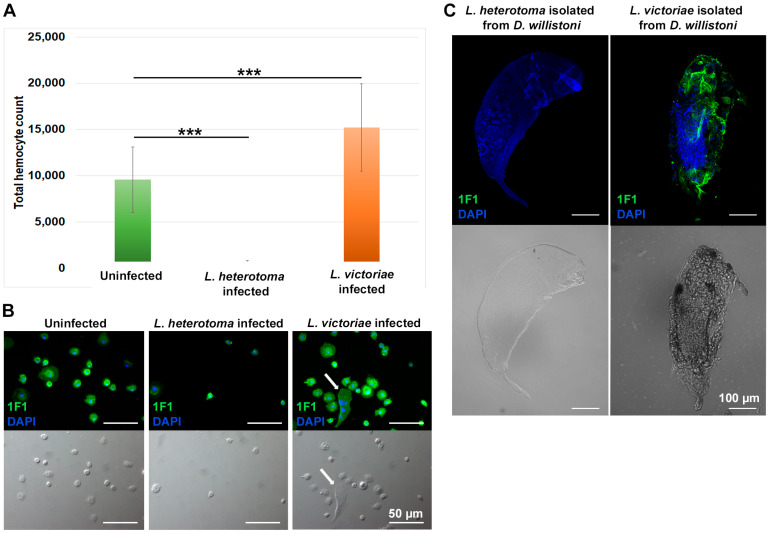
Infection with different parasitoid wasp species influences the total hemocyte count and affects encapsulation ability. Samples were analyzed 72 h post infection. (**A**) Three independent experiments were performed with 12 larvae in each. The error bars indicate the standard deviation. Student’s *t*-test was used for statistical analysis. *p* < 0.001 = ***. (**B**) Visualization of the hemocytes by indirect immunofluorescence using the 1F1 pan-hemocyte monoclonal antibody in uninfected, *L. heterotoma*-infected and *L. victoriae*-infected larvae. The arrow points to a multinucleated giant hemocyte. Staining was analyzed using an epifluorescence Zeiss Axioscope 2 MOT microscope. (**C**) The hemocytes (green) do not attach to *L. heterotoma*, but do attach to *L. victoriae* larvae (blue). Samples were examined with an Olympus FV1000 confocal LSM microscope.

To obtain insights into the population dynamics of hemocytes after infection with *L. heterotoma* and *L. victoriae* parasitoids, we determined the total hemocyte count of the larvae 72 h after parasitization. To visualize the hemocytes, a pan-hemocyte antibody, 1F1 was used. The 1F1 monoclonal antibody reacts with all hemocytes in the circulation of both naïve and *L. victoriae*-infected larvae (Figure 2B). These include small spherical cells, small cells with short extensions and larger flattened hemocytes with diverse morphology. Infection with *L. heterotoma* caused a dramatic decrease in the total hemocyte count, while in *L. victoriae*, there was a significant increase (Figure 2A,B). In *L. victoriae*-infected larvae, hemocytes attached to the parasitoid, while in *L. heterotoma*-infected larvae, they did not attach (Figure 2C).

### 3.3. D. willistoni Hemocytes Characterized by Discriminative Monoclonal Antibodies

**Figure 3:**
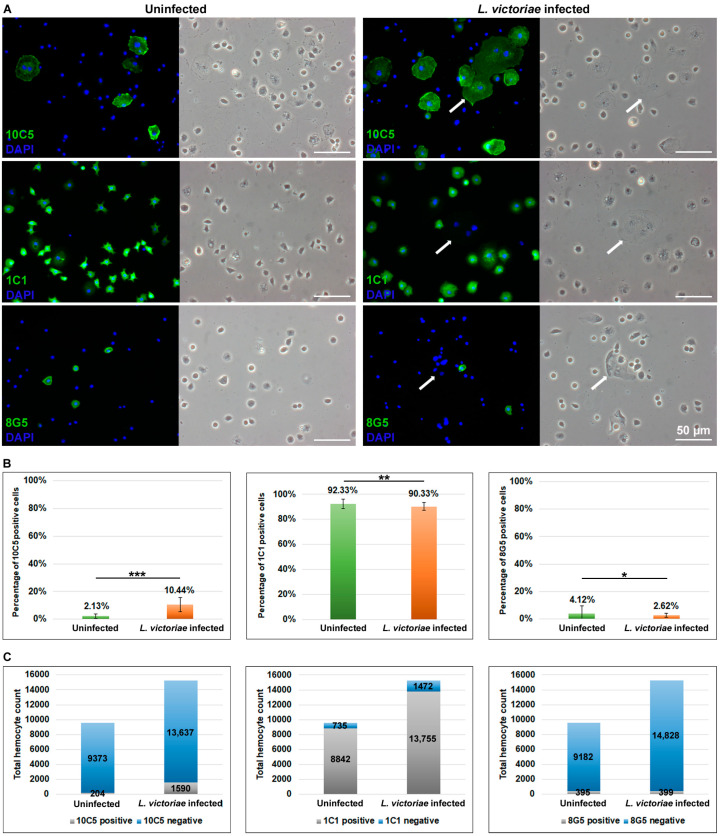
Hemocyte subpopulations in both naïve and *L. victoriae*-infected larvae were identified using discriminative antibodies. Samples of infected larvae were prepared 72 h post infection. (**A**) Hemocyte type specific monoclonal antibodies (10C5, 1C1, 8G5) were used in indirect immunofluorescence experiments to detect the respective hemocyte subpopulations. Arrows point to multinucleated giant hemocytes. Reactions were detected with an epifluorescence Zeiss Axioscope 2 MOT microscope in three independent experiments. (**B**) The ratio of the respective cell subpopulations was related to the corresponding total hemocyte number. Three independent experiments were performed with 24 larvae each. The error bars indicate the standard deviation. Student’s *t*-test was used for statistical analysis. *p*-values: <0.05 = *; <0.01 = **; <0.001 = ***. (**C**) The net hemocyte count of the respective subpopulations. The data of three independent experiments were cropped, with 24 larvae in each.

Hemocytes of *D. willistoni* were previously divided into several classes based on their morphological features [20,21]. To define hemocytes based on expression of immunological markers, we generated and used monoclonal antibodies in combination with functional assays. Three antibodies, 10C5, 1C1 and 8G5, revealed heterogeneity (Figure 3A).

The 10C5 antibody reacted with cells of variable morphology, from spherical to large flattened and, occasionally, binucleated cells in naïve state, while in infected larvae, 10C5 positive hemocytes often became multinucleated (Figure 3A). The parasitoid wasp infection resulted in a 5-fold increase in the proportion of the 10C5 positive subpopulation within the entire hemocyte population (Figure 3B). As the total hemocyte count strongly increased after *L. victoriae* infection (Figure 2A), the net number of the 10C5 positive cells was about eight times more in the infected than in the uninfected samples (Figure 3C).

**Figure 4:**
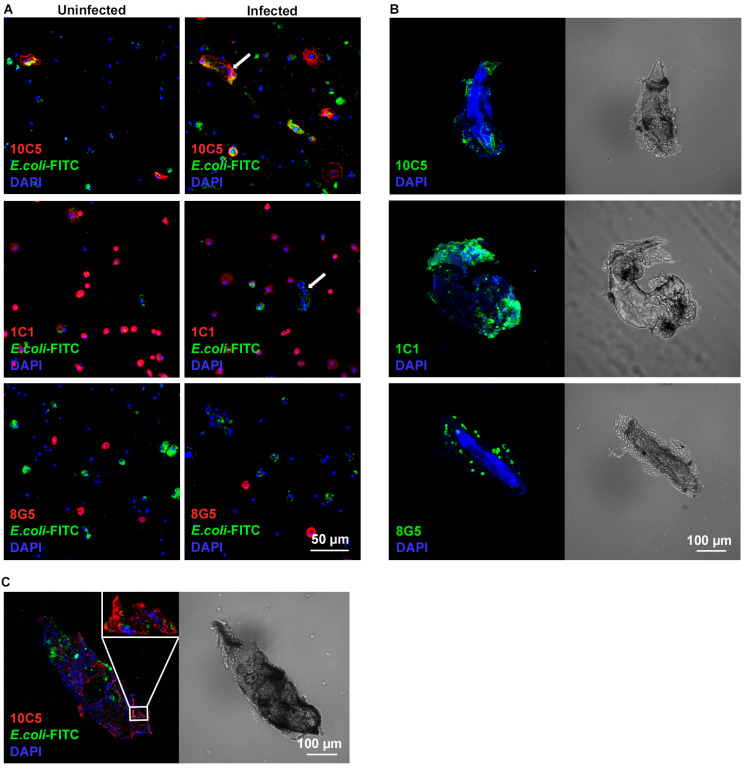
The functional characteristics of the hemocyte subsets. Samples from *L. victoriae*-infected larvae were prepared 72 h post infection. In phagocytosis assays, FITC-labeled *E. coli* bacteria were used. Staining was analyzed with an Olympus FV1000 confocal LSM microscope. (**A**) In vivo phagocytosis of hemocytes in naïve and infected larvae. Two independent experiments were performed, with 12 larvae in each. Images are composed by merging two slides in the nucleus plane. Arrows point to multinucleated giant hemocytes. (**B**) Each hemocyte subpopulation contributed to the encapsulation of the parasitoid wasps. (**C**) 10C5 positive encapsulating cells also phagocytose bacteria.

The 1C1 antibody reacted with 92% and 90% of the circulating hemocytes in the uninfected and infected states, respectively (Figure 3B). The 1C1 negative subpopulation included all large multinucleated cells and mononuclear cells with variable morphology (Figure 3A and Figure 4A). In infected larvae, the proportion of the 1C1 positive hemocytes is slightly reduced (Figure 3B), and the 1C1 negative cell subpopulation, including the multinucleated cells, increased, indicating a shift from the positive subpopulation to the negative subset. Furthermore, as the total hemocyte count increased after *L. victoriae* infection (Figure 2A), the net cell counts in the wasp-infected larvae were elevated in both the 1C1 positive and the 1C1 negative subpopulation (Figure 3C).

The 8G5 antibody reacted with a minor subpopulation composed of small hemocytes (Figure 3A). Following *L. victoriae* infection the proportion of the 8G5 marked cells decreased within the total circulating hemocyte population (Figure 3B); however, based on the strongly increased total hemocyte number (Figure 2A), the net count of the 8G5 positive cells remained stable (Figure 3C).

The 10C5 and 1C1 discriminative antibodies were combined to immunostain hemocytes of *L. victoriae*-infected larvae. These two antibodies covered the whole circulating hemocyte population, as evidenced by the staining of all cells.

### 3.4. Functional Characterization of the Hemocyte Subsets

We first tested, ex vivo, the ratio of phagocytic cells in naïve and *L. victoriae*-infected (72 h post infection) larvae. Fluorescein isothiocyanate (FITC)-labeled *Escherichia coli* bacteria were added to hemocyte isolates. After 1 h incubation, the fluorescence of the non-phagocytosed bacteria was quenched using trypan blue solution, and the ratio of the phagocytic cells was subsequently determined. In both the naïve and the infected larvae, 69.6% of hemocytes phagocytosed bacteria.

Next, phagocytic capacity of the antibody-marked subsets was determined by in vivo phagocytosis assays. Naïve and *L. victoriae*-infected larvae were injected with FITC-labeled *Escherichia coli* bacteria and the phagocytic capacity of the 10C5-, 1C1- and 8G5 positive hemocyte classes was determined. We found that, in both the naïve and the wasp-infected samples, the majority (77%) of the 10C5 positive cells phagocytosed bacteria (Figure 4A). Notably, each multinucleated 10C5 positive cell phagocytosed, which is a novel feature of the so-far-identified encapsulating multinucleated cells in *Drosophilidae*. The 1C1 positive subpopulation included both phagocytic (22%) and non-phagocytic (78%) cells, and their ratio did not change after wasp infection. Interestingly, among the 1C1 negative hemocyte subpopulation, the ratio of the non-phagocytic cells increased after wasp infection from 21% to 39%. The 8G5 positive cells were non-phagocytic (Figure 4A).

The involvement of the antibody-marked hemocyte subsets in the encapsulation reaction was recorded 72 h after infection with *L. victoriae*. Each cell type was present on the parasitoids (Figure 4B), highlighting that they all contribute to the encapsulation reaction. In the cell aggregates settled on the parasitoids melanizing spots were frequently detected (Figure 2C and Figure 4B,C). We observed that both, the encapsulating 10C5 positive and negative cells exhibited phagocytic capacity (Figure 4C).

### 3.5. Differentiation of the 10C5 Positive Cells

**Figure 5:**
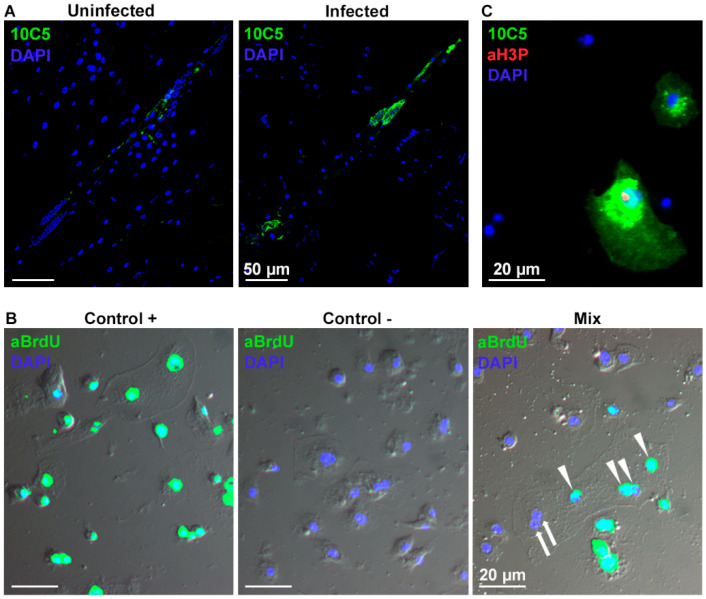
Formation of the 10C5-positive cells. (**A**) The lymph gland contributes to the formation of the 10C5 positive cell subpopulation. Samples were generated from *L. victoriae*-parasitized larvae 24 h after infection and analyzed with an Olympus FV1000 confocal LSM microscope. (**B**) Multinucleated hemocytes are formed by cell fusion. Hemocytes with bromodeoxyuridine (BrdU) labeled (arrowheads) and BrdU-unlabeled (arrows) nuclei fuse and form a multinucleated cell. This was detected with an epifluorescence Zeiss Axioscope 2 MOT microscope. (**C**) Multinucleated cells may develop via nuclear division. Anti-phospho-histone H3 (aH3P) antibody was used to detect the mitotic marker. Analysis was performed with an epifluorescence Zeiss Axioscope 2 MOT microscope.

Following *L. victoriae* parasitoid wasp infection, the most robust changes occurred in the 10C5 positive subpopulation regarding the proportion and number of these cells, including multinucleated hemocytes, (Figure 3B,C). Therefore, we examined whether the main hematopoietic organ, the lymph gland, participates in the generation of this subset. Indirect immunofluorescence analysis of the lymph glands, isolated from third instar naïve larvae, reacted with the 10C5 antibody, showing the presence of the 10C5 specific antigen in several cells, localized predominantly in the middle region of the organ (Figure 5A). This indicates the contribution of the lymph gland to the generation of the circulating 10C5 positive cells of naïve animals. The lymph glands of *L. victoriae*-infected larvae, already at 24 h post infection, carried more 10C5 positive cells than the uninfected samples (Figure 5A), showing that the hematopoietic organ also serves as a source for this cell type in the infected state, and could therefore contribute to the substantial increase in the number of 10C5 positive circulating cells (Figure 3B).

Multinucleation of hemocytes in both *D. ananassae* and *Z. indianus* occurs via cell fusion in circulation [15,22]. Here, using the nucleotide analogue, bromodeoxyuridine (BrdU), which incorporates into the DNA of the cells, we showed that hemocytes of BrdU-treated and BrdU-untreated larvae were able to fuse ex vivo to form multinucleated cells (Figure 5B). Moreover, to verify whether multinucleation can also be the result of nuclear division, we analyzed the presence of the mitotic marker phospho-histone H3 (H3P) in the circulating 10C5 positive cells 48 h after *L. victoriae* infection. Out of 540,000 cells we detected a signal in 0.07% of the hemocytes, but only in 0.0009% of the 10C5 positive cells (Figure 5C), showing that in general, cell fusion (and rarely mitosis) could participate in the formation of multinucleation in these cells.

## 4. Discussion

Insects deploy a continuously developing, robust and complex immune response to keep up with the co-evolution of pathogens. This is manifested in a multifaceted cellular immune response with a substantial morphological and functional diversity of effector cells using a variety of molecules against the pathogens. The immune defense reactions of insects show interesting similarities to those of the mammals; the antimicrobial peptides and proteolytic cascades are regulated by similar regulatory networks, and the effector cells operate on the same principles. The concerted action of the cellular and humoral components of the immune system provides prompt elimination of microbes by phagocytic cells and encapsulation of larger particles by specialized cell types transdifferentiating from phagocytic cells [1,2,3]. Lamellocytes have been described in numerous species of the so-called “oriental” subgroups of the *melanogaster* group, including the *melanogaster*, *eugracilis*, *suzukii*, *ficusphila*, *elegans*, *rhopaloa* and *oshimai* subgroups within the *Drosophilidae* family [14]. A combination of antigen expression analysis with functional assays has defined a variety of other encapsulating cell types in the species of the *ananassae* subgroup [15] and in *Zaprionus indianus* [22]. These species possess a network of elongated, multiform cells and anucleated cell fragments, which are involved in the formation of multinucleated syncytia with variable morphological features by homotypic cell–cell fusion [15,16,22]. All these species have a highly efficient immune defense reaction against parasitoids [15,17]. In this work, we describe another multinucleated encapsulating cell type in *D. willistoni*, which possesses a binary function, as these cells are also phagocytic. Previously, hemocytes of *D. willistoni* have been classified based on their distinctive morphological features [20], but here, we combined immunological and functional assays to follow the dynamics and role of hemocyte subpopulations in the naïve state and following parasitoid infections.

First, we analyzed the defense against three closely related parasitoid wasp species, *L. victoriae*, *L. heterotoma* and *L. boulardi*, and found that only the first two parasitized *D. willistoni* larvae, while *L. boulardi* was not attracted. The fact that *L. boulardi* wasps readily attacked a mixture of *D. melanogaster* and *D. willistoni* larvae suggested that the lack of parasitization was due to an absence of sensing and not by a repellent substance produced by *D. willistoni*. Both, *L. victoriae* and *L. heterotoma* inject venom into the hosts to block effective immune responses and the killing of their larvae; however, there are substantial differences in the effect of the venom [23,24,25]. We showed that *L. heterotoma* infection suppressed the immune response as it caused lysis of hemocytes. *L. victoriae* infection stimulated the immune reaction as infection induced a substantial increase in the total hemocyte count and did not affect hemocyte activation or the successful encapsulation reaction in *D. willistoni*. In contrast, we previously showed that oviposition of *L. victoriae* actively suppressed the immune reaction of *D. ananassae* as it caused a strong decrease in hemocyte count [15]. These two species show different abilities to encapsulate the same parasitoid, *L. victoriae*, which suggests that the co-evolution of parasites and hosts in different geographical regions evolved differently. The native range of *D. ananassae* is South and Southeast Asia and the Indo and South Pacific [26], while *D. willistoni* geographically originates from South America [27]. The parasitization assays also highlighted a more effective immune defense in *D. willistoni* than in *D. melanogaster* against both *L. heterotoma* and *L. victoriae* (Figure 1B).

The immune defense reaction of *D. willistoni* to *L. victoriae* was further characterized by the use of discriminative monoclonal antibodies. The cell subpopulation, which showed an impressive shift after parasitization with *L. victoriae*, reacted with the 10C5 antibody. These were larger, flattened cells that were also present in the naïve state, and the subpopulation was mainly composed of mononuclear cells. After *L. victoriae* infection, the ratio of the 10C5 positive cells showed an extensive increase, and multinucleated cells differentiated (Figure 3 and Figure 4A). The multinucleated cells all expressed the 10C5 antigen and lacked the 1C1 antigen. Similarly, to the MGHs of *D. ananassae* and *Z. indianus*, we found that the 10C5 positive hemocytes of *D. willistoni* settled on the parasitoid, suggesting their involvement in the capsule formation and anti-parasitoid defense reactions (Figure 4B,C). Furthermore, differentiation of the MGHs varies across species because, while MGHs are formed by cell fusion during circulation in *D. ananassae* and *Z. indianus* [15,22], in *D. willistoni*, nuclear division could also be involved in the formation of these cells (Figure 5C). Although mitotic events were detected in the 10C5 positive cells, we observed that cell fusion was a more frequent phenomenon for differentiating MGHs.

Surprisingly, we found that, in both naïve and parasitoid wasp-infected *D. willistoni* about 30% of the hemocytes were non-phagocytic, while in *D. melanogaster* and in *Z. indianus*, the ratio of these cells was about 5% of the total hemocyte population [11,12,22]. The role of the large number of non-phagocytic cells in *D. willistoni* is not clear. This group of cells also includes the 8G5 positive subpopulation, but the 8G5 positive cells only represent a maximum of 4% of the hemocytes (Figure 3B), which means that there are other non-phagocytic cells in about 26%, which do not carry the 8G5 specific antigen. We found that, among the 1C1 negative hemocyte subpopulation, the ratio of the non-phagocytic cells doubled after *L. victoriae* infection, suggesting that this group of hemocytes might have acquired another defense function while losing their phagocytic ability. Furthermore, we found that the majority (about 77%) of the 10C5 positive cells, including all multinucleated cells, phagocytosed bacteria (Figure 4), while MGHs of *D. ananassae* and *Z. indianus* and lamellocytes of *D. melanogaster* were non-phagocytic [15,22]. We found that 10C5 positive cells of *D. willistoni*, including multinucleated hemocytes, kept their phagocytic capacity (Figure 4B) even when localized on the parasitoid wasp (Figure 4C). Similar to the 10C5 positive cells of *D. willistoni*, in three species of the *obscura* group, *Drosophila tolteca*, *Drosophila affinis* and *Drosophila obscura*, pseudopodocytes differentiated to encapsulate parasitoids, and they also kept their phagocytic ability [28,29]. It is not clear yet whether the binary function of the 10C5 positive cell type of *D. willistoni* serves as an evolutionary advantage, contributing to a highly effective, multifaceted cellular immune response. In conclusion, the large number of non-phagocytic cells in *D. willistoni* may explain why the 10C5 positive hemocytes, including the multinucleated cell population, maintained their phagocytic ability and hence retained the number of the phagocytic cells at a respectable level.

*D. willistoni* has evolved a more effective defense reaction against *L. victoriae* and *L. heterotoma* parasitoids than *D. melanogaster* (Figure 1B). The genome of *D. willistoni* does not have an ortholog of PPO3, a gene specifically associated with lamellocytes which are restricted to a limited number of drosophilids and essential for melanization in the capsule in these species [10,14]. This observation suggests that *D. willistoni* has evolved a distinct defense mechanism against parasitoids. However, we observed partial, spotty melanization in the capsule (Figure 2C and Figure 4B,C). Although *L. heterotoma* infection caused a drastic decrease in the number of circulating hemocytes (Figure 2A,B) and the cells did not attach to the developing wasp larvae (Figure 2C), some adult flies eclosed after wasp infection, suggesting the involvement of humoral defense factors in anti-parasitoid immunity. We found that the *D. willistoni* LOC124460379 gene (NCBI database) encodes for a Hemolysin E-like protein (XP_046866967.1), and another gene, localized 4 kb upstream of the LOC124460379, which has not been annotated in GeneBank, encodes for a homolog protein, showing 64% identity with XP_046866967.1. Hemolysin E proteins are pore-forming toxins produced by certain microbial species belonging to the *Enterobacteriaceae* family and used to attack eukaryotic cells [30,31,32]. Such genes were detected in *D. ananassae* and are supposed to be involved in the elimination of parasitoids [33]. Moreover, other genes captured by horizontal gene transfer encode for humoral factors produced by the fat body and are functional modules in the anti-parasitoid defense of *D. ananassae* subgroup species [34]. The possible presence of a humoral anti-parasitoid immune defense reaction in *D. willistoni* is also supported by the observation that this species is resistant to *Asobara tabida* parasitoid infection through mechanisms other than encapsulation [21].

This study provides insight into the immune defense reactions of *D. willistoni*, involving multinucleated effector cells, which are able to both encapsulate parasitoid wasps and phagocytose bacteria. The high ratio of circulating non-phagocytic cells indicates that hemocyte subpopulations with alternative functions could also be involved in the efficient anti-parasitoid defense reaction. These data extend our knowledge and highlight evolutionary advantages regarding hemocytes of *Drosophilidae*.

## Data Availability

The data presented in this study are available from the corresponding author upon request.
